# Evaluation of two dairy herd reproductive performance indicators that are adjusted for voluntary waiting period

**DOI:** 10.1186/1751-0147-54-5

**Published:** 2012-01-30

**Authors:** Emma Löf, Hans Gustafsson, Ulf Emanuelson

**Affiliations:** 1 Department of Clinical Sciences, Division of Ruminant Medicine and Veterinary Epidemiology, Swedish University of Agricultural Sciences. P.O. Box 7054, SE-750 07 Uppsala, Sweden; 2 Swedish Dairy Association, P.O. Box 210, SE-101 24 Stockholm, Sweden; 3 Department of Clinical Sciences, Division of Reproduction, Swedish University of Agricultural Sciences, P.O. Box 7054, SE-750 07 Uppsala, Sweden

**Keywords:** Cattle, dairy, reproductive performance, voluntary waiting period

## Abstract

**Background:**

Overall reproductive performance of dairy herds is monitored by various indicators. Most of them do not consider all eligible animals and do not consider different management strategies at farm level. This problem can be alleviated by measuring the proportion of pregnant cows by specific intervals after their calving date or after a fixed time period, such as the voluntary waiting period. The aim of this study was to evaluate two reproductive performance indicators that consider the voluntary waiting period at the herd. The two indicators were: percentage of pregnant cows in the herd after the voluntary waiting period plus 30 days (PV30) and percentage of inseminated cows in the herd after the voluntary waiting period plus 30 days (IV30). We wanted to assess how PV30 and IV30 perform in a simulation of herds with different reproductive management and physiology and to compare them to indicators of reproductive performance that do not consider the herd voluntary waiting period.

**Methods:**

To evaluate the reproductive indicators we used the SimHerd-program, a stochastic simulation model, and 18 scenarios were simulated. The scenarios were designed by altering the reproductive management efficiency and the status of reproductive physiology of the herd. Logistic regression models, together with receiver operating characteristics (ROC), were used to examine how well the reproductive performance indicators could discriminate between herds of different levels of reproductive management efficiency or reproductive physiology.

**Results:**

The logistic regression models with the ROC analysis showed that IV30 was the indicator that best discriminated between different levels of management efficiency followed by PV30, calving interval, 200-days not-in calf-rate (NotIC200), in calf rate at100-days (IC100) and a fertility index. For reproductive physiology the ROC analysis showed that the fertility index was the indicator that best discriminated between different levels, followed by PV30, NotIC200, IC100 and the calving interval. IV30 could not discriminate between the two levels.

**Conclusion:**

PV30 is the single best performance indicator for estimating the level of both herd management efficiency and reproductive physiology followed by NotIC200 and IC100. This indicates that PV30 could be a potential candidate for inclusion in dairy herd improvement schemes.

## Background

The overall reproductive performance of dairy herds is monitored by various measurements and indicators. In Sweden those measurements are computed in the Swedish Official Milk Recording Scheme (SOMRS). SOMRS is a voluntary service for dairy herds and is an equivalent of a dairy herd improvement program (DHI) in the USA. In Sweden 80% of the herds are enrolled to the SOMRS and they have regular access to the information on how they perform, both in relation to their own historical records and for benchmarking against other herds. Some herds consult an advisor to help them analyze and prioritize what should or could be done regarding to the information that they are given from the SOMRS. In Sweden there is a policy not to use oestrous synchronization and timed artificial insemination (AI) for dairy cows. Most cows (approx.85%) are subjected to AI and the herds practice year round calving.

When monitoring the reproductive performance, time interval measurements are frequently used as reproductive performance indicators and many are calculated in relation to the cow's individual calving date. Examples of such indicators are the calving interval (CI), days to first service (CFI) and days to conception or last insemination (CLI). The drawbacks with these indicators are that they can only be calculated for animals that either have a consecutive calving or have been inseminated and/or checked for pregnancy (depending on indicator), thus introducing possible selection bias. Cows that have not been inseminated or cows that fail to conceive or to calve again are never included in those kinds of indicators and therefore they are not completely representative of a herd's reproductive status. This problem can be alleviated by measuring the proportion of pregnant cows by specific intervals after their calving date or after a fixed time period and using survival analysis on the time-to-event data, where all information, also on animals without the event, is used. The 100-days in calf-rate (IC100) is an increasingly popular indicator that uses this methodology [1,2].

However, most indicators do not consider the different management strategies applied at farm level, such as the herd's voluntary waiting period (VWP). The VWP is the time period between calving and when the management of the herd decides the cow is ready for breeding and it gives the cow some time to resume normal ovarian cyclicity. This time period might be decided in advance by the farmer's management strategy and in Sweden it is suggested by herd advisors to be 50-60 days in milk for the herd. An American study [3] found that the VWP, as reported by the herdsmen, for 673 American dairy herds had a mean of 55.6 ± 0.6 days with a range between 30 and 90 days. Similar numbers have also been found in Sweden where the VWP, as reported by the herdsmen, had a mean of 66.5 days with a range between 50 and 80 days [4]. The VWP obviously varies between herds, but may also vary within a herd according to the cow's parity and milk yield [5]. With such variable VWPs, the commonly used reproductive indicators are largely influenced by strategic or managerial decisions along with biological variation and it can be difficult to compare the reproductive efficiency between herds with different management strategies. Consequently, the commonly used indicators do not only reflect the biological reproductive performance of the cow. A way to reduce the variation caused by herd management and to better reflect the biological reproductive performance is to use an indicator that controls for VWP at the herd level. The variation in VWPs will also influence the genetic evaluation of dairy sires for daughter fertility. A proposed model [6] for the calculation of the American breeding value for daughter pregnancy rate includes both censoring of records from non-pregnant cows and calculated herd specific VWP.

Herd management and the reproductive physiology are reflected differently by various reproductive indicators. Depending on the target of the monitoring, i.e. the management level or the reproductive physiology, different performance indicators might be needed. The best indicator to measure the physiological reproductive performance is not necessarily the best indicator to measure the reproductive management level.

Evaluating reproductive performance indicators on data from actual herds may be problematic because the "true" status is never known and it is difficult to know what component and to what extent that component has influenced the reproductive status of that herd. The inherent natural variation of the components that influences the reproductive status makes it necessary to acquire a large amount of data to evaluate differences between herds. To control the setting one could perform large scale experiments, but they are both expensive and time consuming. Stochastic simulations have therefore been used to exemplify herds with different reproductive status [7,8]. Simulation studies of economic consequences of postponed first insemination in herds with different reproduction management [9] and different times for the start of inseminations in herds with different culling rates [10] have been carried out previously. Successful reproduction, i.e. pregnancy, relies on complex physiological dynamics and is the result of a chain of events. The resumption of ovarian cyclicity, oestrus and ovulation are all events that need to precede conception and a failure at one stage results in a failure in the whole process [11]. The animal and its organs are influenced by both internal (genotype, parity and milk production) and external (nutritional and management) factors. All these multiple factors can be integrated in mathematical modelling to characterize an animal's reproductive state efficiently [12].

The aim of this present study was to assess how two reproductive performance indicators, which are adjusted for the herd VWP and based on survival analysis, perform in a simulation of herds with different reproductive status and how they compare to traditionally used reproductive performance indicators that do not consider the VWP. The two indicators were percentage of pregnant cows in the herd after the herd voluntary waiting period plus 30 days (PV30) and percentage of inseminated cows in the herd after the herd voluntary waiting period plus 30 days (IV30).

## Methods

### Description and outline of the simulation model

#### SimHerd

To obtain herds with different reproductive status we used the SimHerd model, which is available for research purposes (http://www.simherd.com). SimHerd is a dynamic, stochastic and mechanistic model that predicts the production and states of a dairy herd over time by simulating changes by weekly time steps [13]. The unit of simulation is the individual cow and the state of the cow's feed intake, milk yield, disease occurrence, reproduction and mortality are simulated. For example, when regarding reproduction, discrete events, such as oestrus, heat detection, conception, foetal death, disease, non-voluntary culling and milk yield potential, are triggered stochastically using random numbers from relevant probability distributions [14,15]. Replacement heifers are recruited from own young stock if available, otherwise through purchase of pregnant heifers. Voluntary culling decisions are based on the number of days open and milk yield. Non-voluntary culling (i.e. death) accounts for cullings triggered by unforeseen events. SimHerd requires an initial herd that consists of a list of cows and heifers that are described by their state variables, as described above, at a certain date. On this initial herd one can alter some decision variables that influence the development for the herd. We wanted to produce an initial herd with a maximum of 100 cows and a minimum of 90 cows; the anticipated herd size for a future Swedish herd. We also defined a number of decision variables for altering reproductive parameters (as described below) and used scenario 5 (i.e. an average herd with short VWP, see Table 1) to produce an initial herd and simulated this herd for 20 years to have a stable starting-point for our simulated scenarios. The generated average annual milk yield for the last 5 years of simulation was 9746 kg of ECM per cow and the average herd size was 98.8 cows.

**Table 1:** Numeric identification of the 18 different scenarios with combinations of voluntary waiting period (VWP), reproductive management efficiency and reproductive physiology

		Reproductive physiology		
		
VWP	Management	Good	Average	Poor
Short	Good	1	2	3
	Average	4	5	6
	Poor	7	8	9
Long	Good	10	11	12
	Average	13	14	15
	Poor	16	17	18

#### Simulated scenarios

We simulated 18 different scenarios, which can be seen as 18 different types of herds. Each scenario was simulated over 10 years with 50 replications, which can also be seen as 50 different herds within each scenario. The data from the two last years of simulation were used for the statistical analysis and for comparing the reproductive indicators. The rationale for using the two last years was that enough time has been allowed for the simulation to be stabilised and avoiding the random variation of a single year.

#### Decision variables

In SimHerd, there are decision variables related to the reproductive performance and they can be altered to model the reproductive status of the cow. Of these, some are related to the reproductive strategy or management of the herd, such as oestrus detection and when to start breeding, whereas some are related to different constraints in the reproductive physiology, such as conception and abortion rates. The different combinations of VWP, reproductive management efficiency and reproductive physiology for the different scenarios are shown in Table 1 and are described below. Decision variables, that were time intervals, were multiples of 7 because the SimHerd-program uses weekly time increments. The range of intervals and different rates and percentages were based on soliciting views and experiences of reproductive experts.

### Levels of decision variables used in the simulations

#### Voluntary waiting period

Two different herd management strategies to determine when to start the insemination period were used in the simulations. Short VWP was defined as starting inseminations at 49 days *post partum *(scenario 1-9) and long VWP as 77 days (scenario 10-18).

#### Reproductive management efficiency

Different efficiencies were defined using different oestrus detection rates and different length of time between insemination and pregnancy checks (PC). Three levels of reproductive management efficiency were used in the simulations: Good, Average and Poor. Good efficiency was defined as 80% heat detection and PC after 56 days; Average efficiency as 50% heat detection and PC after 70 days and Poor efficiency was 30% heat detection combined with PC after 84 days.

#### Reproductive physiology

In order to define the status of reproductive physiology of the different herds we decided to alter embryonic death and foetal loss and conception rate (CR). Three levels, Good, Average and Poor, of reproductive physiology were constructed combining three different levels of embryonic death and foetal loss with three different CR. Good reproductive physiology was defined as 20% loss and 60% CR; Average as 25% loss and 45% CR and Poor was defined as 30% loss and 35% CR.

### Reproductive performance indicators

We calculated herd means for several reproductive performance indicators based on data from the last two years in each simulated scenario. The different indicators were CFI, CLI, CI, fertility index (FI), IC100, 200-day not-incalf-rate (NotIC200), PV30 and IV30. The calculations for each indicator are described below.

CFI and CLI are measured in days and are only calculated for cows that have had at least one calving and one subsequent insemination. CI is also measured in days and is only calculated for cows that have had at least two calvings. FI is a composite index that is used in the Swedish DHI. It is modified from de Kruif [16] and combines several measures into one and is aimed to give the farmer an overall idea about the fertility level of the herd. The equation for FI in the Swedish DHI system is as follows:

$$\begin{array}{c} FI=\left(\frac{\left(\%NR56-\left(CLI-125\right)\right)}{NINS}\right)\times \\ \left(\frac{\left(Number\;of\;animals\;submitted-Withdrawn\;animals\right)}{Number\;of\;animals\;submitted}\right) \end{array}$$

In the equation above, "%NR56" is the non-return rate at 56 days after breeding and it is calculated as the proportion of cows not rebred in 56 days after an insemination. "NINS" is the average number of inseminations per cow in the herd. The "number of animals submitted" is calculated as all cows eligible and chosen for inseminations. "Withdrawn animals" are, in the original equation, animals that have been withdrawn or culled because of reproductive failure or problems. In the SimHerd-program, reasons for cullings are not given and we choose therefore to let 25% of the total number of cullings be attributed to reproductive failure or problems, which is the average proportion observed under Swedish conditions [17].

PV30 and IC100 are the proportions of pregnant cows at the herd 30 days after the herd VWP and 100 days after calving, respectively. IV30 is the proportion of inseminated cows at the herd 30 days after the herd VWP. NotIC200 is the proportion of non-pregnant cows 200 days after calving. These four indicators are all estimated using survival analysis (as described below) and account for potential censoring, i.e. cows culled before having conceived or before a first insemination.

The reason for choosing 30 days after the VWP, for the indicators PV30 and IV30, was based on a normal oestrus cycle length of 21 days, but with an addition of a few days allowing for the fact that the VWP is not a definite length of time and that the cycle length might vary between cows.

### Statistical analysis

All statistical analyses were performed using the software package SAS (version 9.1, Cary, N.C., USA). To calculate PV30, IV30, IC100 and NotIC200, survival analysis was performed using Cox's proportional hazards regression model by using the PHREG procedure with no predictor variable but with days to pregnant or days to insemination as the response variable. Censoring of cows was done at the day of death or removal from the herd or at the last day of simulation if not removed, not pregnant or not inseminated. The survivor function estimate gave the proportion of pregnant or inseminated cows at a given day and for PV30 and IV30, the survivor function estimate was read at 79 days (i.e. VWP of 49 days + 30 days) for scenario 1-9 and at 107 days (i.e. VWP of 77 days + 30 days) for scenario 10-18. The survivor function estimate was read at 100 days for the indicator IC100 for all scenarios and at 200 days for the NotIC200.

Logistic regression models were used to examine to what extent the different reproductive performance indicators could discriminate between herds of different levels of reproductive management efficiency or reproductive physiology. That is, the predictive ability of the model was evaluated by receiver operating characteristics (ROC) analysis using the LOGISTIC procedure with the ROC option. Reproductive performance indicator was the only explanatory effect in the model. The levels of reproductive management efficiency and reproductive physiology (see Table 1 for reference) were dichotomized, where Good was classified as one group, and Average and Poor were classified into the other group, in order to simplify the comparisons. This was done separately for reproductive management efficiency and reproductive physiology. The group Good reproductive management efficiency consisted of the herds in scenario 1, 2, 3, 10, 11 and 12, while the group Average+Poor reproductive management efficiency consisted of the herds in the remaining scenarios. The group Good reproductive physiology consisted of the herds in scenario 1, 4, 7, 10, 13, 16 and the group Average+Poor reproductive physiology consisted of the herds in the remaining scenarios.

## Results and discussion

### Reproductive performance indicators

**Figure 1:**
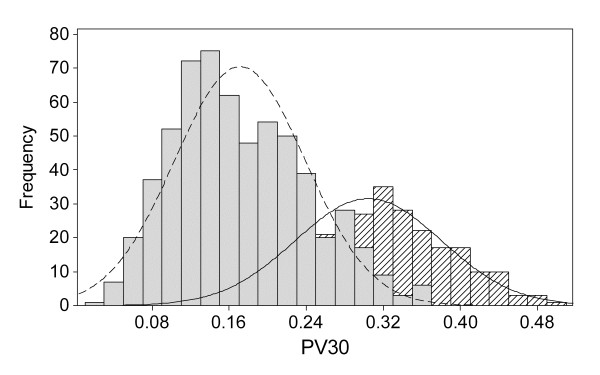
**Histogram of herd means for the reproductive indicator PV30**^a^**divided into two groups of different management efficiency, Good (Diagonal lines, N = 300) and Average+Poor (Filled grey, N = 600)**. ^a^PV30 = proportion of pregnant cows 30 days after the herd VWP.

**Figure 2:**
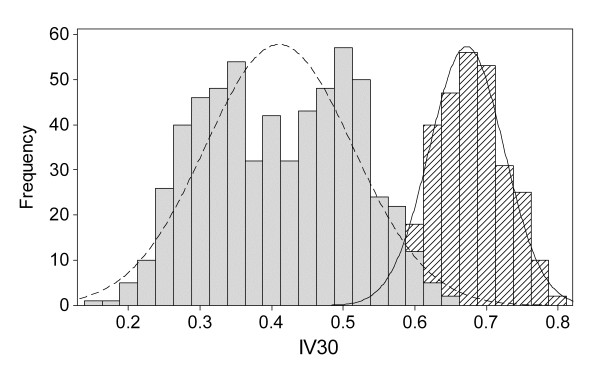
**Histogram of herd means for the reproductive indicator IV30**^a ^**divided into two groups of different management efficiency, Good (Diagonal lines, N = 300) and Average+Poor (Filled grey, N = 600)**. ^a^IV30 = proportion of inseminated cows 30 days after the herd VWP.

The distributions for some of the reproductive performance indicators overlap considerably in the different scenarios. This is illustrated in Figure 1 where the distribution of PV30 in two groups of simulated reproductive management efficiencies is shown. The mean of PV30 in the Good group (mean = 0.304 SD = 0.076) is higher compared to the Average+Poor group (mean = 0.171 SD = 0.068) but there is no clear threshold for the value of PV30 at which the Average+Poor group is distinguished from the Good group. If one compares this to the distribution of IV30 in two groups of simulated management efficiency (Figure 2) there is a clearer threshold where the groups can be distinguished from each other. The mean value for IV30 in the Good group was 0.673 with a SD of 0.050 and in the Average+Poor group the mean was 0.410 and a SD of 0.104. This means that there would be a larger proportion of Good herds misclassified as belonging to the other group for PV30 than for IV30.

### Reproductive management efficiency

**Figure 3:**
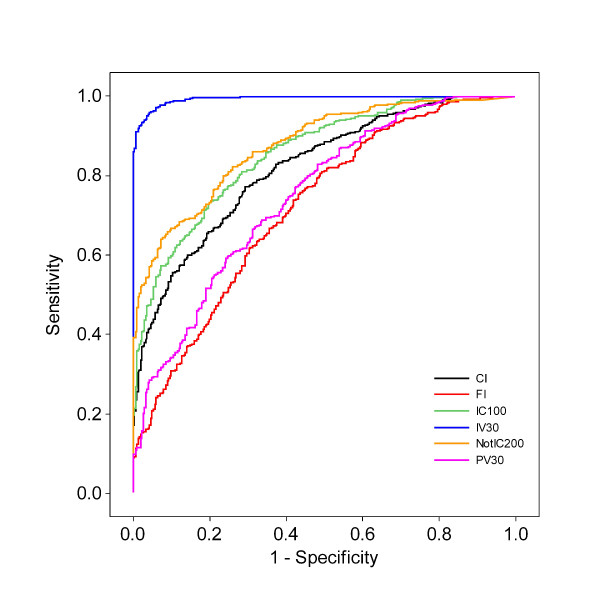
**Receiver operating characteristic curves from the logistic regression model where different reproductive performance indicators predicted the outcome of management efficiency**. CI = Calving interval, days. FI = fertility index. IC100 = proportion of pregnant cows 100 days after calving. NotIC200 = proportion of non pregnant cows 200 days after calving. PV30 = proportion of pregnant cows 30 days after the herd VWP. IV30 = proportion of inseminated cows 30 days after the herd VWP.

The ROC analysis tested which of the reproductive performance indicators that best discriminates between two groups with different reproductive management efficiency. A ROC-curve is obtained by calculating the sensitivity and specificity of every observed data value and plotting sensitivity against 1-specificity. The area under the ROC curve (AUC) can be interpreted as the probability that a herd in the Good group has a higher (or lower when concerning CI and NotIC200) value for the performance indicator of interest than a herd in the Average+Poor group. The results are shown in Figure 3 and Table 2. The IV30 had the greatest AUC followed by PV30, CI, NotIC200, IC100 and the FI. Given that the IV30 had the greatest AUC, IV30 is suggested to be the best indicator when predicting the herd's reproductive management efficiency as defined in the simulation. This can also be seen in the histograms in Figures 1 and 2, where the distribution of IV30 provides a clearer threshold between the two groups than for the PV30. The distributions for the two groups are almost separated for the performance indicator IV30, which ranges from almost zero to around 65% in the Average+Poor group and from 50% to nearly 90% in the Good group. As expected, if the heat detection rate is high more animals are inseminated earlier and the IV30 will probably be more directly affected than the PV30 since more factors influence whether a cow becomes pregnant than whether a cow will be inseminated or not. The outcomes for the different scenarios will thus be less distinct for the PV30 than for the IV30, as would also be the case for the other indicators.

**Table 2:** Results from the Receiver Operational Characteristics (ROC) curve from the logistic regression model showing the area under curve and ranking for the different reproductive performance indicator divided on management efficiency and reproductice performance

	Reproductive performance indicator	Area under curve	95% Wald Confidence Limits	Rank
**Management efficiency**	CI^a^	0.877	0.794-0.849	5
	FI^b^	0.743	0.777-0.833	6
	IC100^c^	0.897	0.851-0.896	3
	NotIC200^d^	0.886	0.885-0.925	4
	PV30^e^	0.902	0.895-0-931	2
	IV30^f^	0.999	0.990-0.996	1
**Reproductive physiology**	CI^a^	0.663	0.627-0.699	5
	FI^b^	0.823	0.794-0.852	1
	IC100^c^	0.707	0.673-0.742	4
	NotIC200^d^	0.744	0.711-0.777	2
	PV30^e^	0.727	0.694-0.760	3
	IV30^f^	0.511	0.471-0.551	6

^a^CI = Calving interval, days

^b^FI = fertility index

^c^IC100 = proportion of pregnant cows 100 days after calving

^d^NotIC200 = proportion of non pregnant cows 200 days after calving

^e^PV30 = proportion of pregnant cows 30 days after the herd VWP

^f^IV30 = proportion of inseminated cows 30 days after the herd VWP

### Reproductive physiology

**Figure 4:**
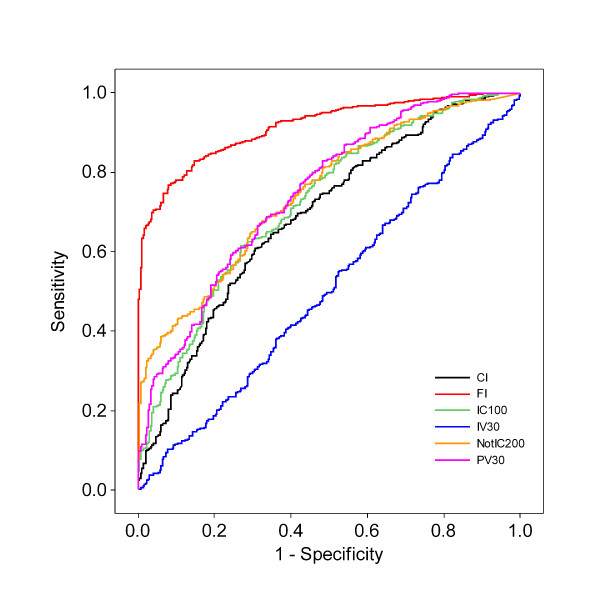
**Receiver operating characteristic curves from the logistic regression model where the different reproductive performance indicators predicted the outcome of reproductive physiology**. CI = Calving interval, days. FI = fertility index. IC100 = proportion of pregnant cows 100 days after calving. NotIC200 = proportion of non pregnant cows 200 days after calving. PV30 = proportion of pregnant cows 30 days after the herd VWP. IV30 = proportion of inseminated cows 30 days after the herd VWP.

The results from evaluating which of the reproductive performance indicators that best could discriminate between two groups with different reproductive physiology are shown in Figure 4 and Table 2. The FI had the highest AUC followed by PV30, NotIC200, IC100 and CI. IV30 could not discriminate between the two groups. Providing that the FI had the greatest AUC, FI is suggested to be the best performance indicator to evaluate the herd's reproductive physiology status. In the simulations the status of reproductive physiology was modified by changing embryonic death and foetal loss and CR. These factors might have affected the FI more than they affected PV30, where the alterations did not have such a large effect on the number of cows that became pregnant within the stipulated time frame. The way the FI is calculated might make it more sensitive to changes in the reproductive physiology and therefore create a wider spread in the value for the indicator and so make it easier to discriminate between the two groups, Good and Average+Poor. That the IV30 could not discriminate between the two groups might not be that surprising since the conception rate and embryonic and foetal losses do not affect whether the animals get inseminated in the first place. The animal has to be inseminated before the event of loss or before it can conceive.

### Evaluation of PV30 and IV30

A main part of differences in reproductive performance between herds is probably caused by differences in management at herd level and not by systematic differences in the reproductive physiology of the individual cows. That is, herd level factors can potentially have larger effects on the reproductive performance, measured at herd level, than factors acting on individual cows [18]. It is therefore more important to have a performance indicator that is good at revealing the management efficiency. Fricke *et al*. [19] found that heat detection efficiency, i.e. a factor influenced by management, had the largest potential impact on the reproductive performance since it is more easily improved compared to conception risk, i.e. a factor largely decided by physiology rather than management. Combining our results on management efficiency and reproductive physiology, the single best indicator seems to be PV30 because it has high AUC in both cases, but the final appraisal depends clearly on the target for the monitoring. For example, the FI was better than PV30 at estimating the status of reproductive physiology while the IV30 was better at estimating the management efficiency, but could not be used to estimate the reproductive physiology since it could not discriminate between the different groups of reproductive physiology. A low value for IV30 indicates that a low proportion of cows at the herd have had their first insemination at VWP+30 days and a low value for PV30 tells us that a low percentage of cows in the herd are pregnant by VWP+30 days. IV30 indicates whether the herdsman has tried to get the cows pregnant by starting to inseminate them. The PV30 says more on how well he has achieved in getting them pregnant. That the cows have had one insemination is more dependent on management than on physiology/biology since they need to be inseminated in order to become pregnant. In this way PV30 and IV30 can be used together. If the PV30 has a low value in the herd and IV30 also has a low value the reason can be that few cows have been inseminated. If the PV30 is low and IV30 is high the herdsman will have trouble in getting cows pregnant, maybe because of wrong timing.

Both IC100 and NotIC200 turned out to be almost as good as the PV30 when ranking the indicators in the ROC-analysis, although they did not adjust for the herd VWP which we believe is an advantage of PV30. However, we used only two lengths of VWP in the simulation, with a rather small difference (49 days *versus *77 days), and effects may have been larger if we had chosen VWPs with more varied time lengths. Stratifying the analyses by VWP gave similar results, i.e. the ranking of the indicators in the ROC analysis was the same (results not shown).

Even though most studies show that a length of the CI of 12-12.5 months is economically optimal [9,20,21], there is an ongoing discussion regarding the benefits of voluntarily extending the CI by increasing the VWP. Increasing the VWP with 60 days in high yielding cows was found to have economic advantages [22]. Österman and Bertilsson [23] concluded that a prolonged CI (VWP = 280 days), combined with milking three times a day, gave a more tenable production system than a conventional one (VWP = 50 days). There is an apparent ongoing trend where more herds vary their VWP. For example, the range of VWP reported by DeJarnette [3] is wide, 30 to 90 days. The prolonged VWP is something that some farmers may approve of and may try to implement at farm level, and it is therefore conceivable that it may be useful for the advisors in the DHI to have a reproductive performance indicator that considers the VWP. The actual VWP at farm level may not be readily available without asking the herdsman, which is not practically possible when using the indicator on a national level. An alternative is to calculate when 5% or 10% [24] of the animals in a herd have had their first insemination. We have previously investigated the correlation between the VWP, as reported by the herdsman, and the calculated time as to when 5% of the cows were inseminated and found an overall correlation of 0.51 [25], which is rather low and suggests that herdsmen either do not have a defined strategy or that the VWP is not strictly implemented, maybe because of the use of individual VWPs. A calculated VWP may therefore be more reasonable to use than the VWP given by the farmer when calculating reproductive performance indicators that considers the VWP.

Some bias in reproductive performance indicators are avoided by considering VWP and by applying survival analysis in calculating PV30 and IV30. However, animals that are not intended to be inseminated will be included in the calculation and this proportion may vary between herds and thus introduce some bias. The same concern applies, however, also to some of the other indicators such as IC100 and NotIC200.

### Validation of the simulation

The results obtained in this study rely on simulated data, which reflects an "ideal world" where all information on the events is complete and correct. This is seldom the case in the "real world" where, for instance, not all animals are pregnancy checked after every insemination and data can be lost or misread. However, loss of data or imprecise data affects all indicators and we have no reason to believe that the ranking of the performance indicators would be affected. Results based on simulated data are also confined by the simulation environment and the parameters that can be modified. This may affect the precision of the estimates and possibly also the ranking of the indicators. The alternative, to work on real, observed data, is clearly also affected by the underlying mechanisms, which cannot be observed or controlled in an experiment, and it is therefore not a better alternative to objectively assess the relative merits of different reproductive performance indicators than using simulated data.

For each simulated scenario the mean value and SD over the 50 simulations were calculated for each of the reproductive performance indicators. The results for scenario 10-18 are presented in Table 3. Scenario 1-9 produced similar results, but had of course shorter intervals due to the shorter VWP. We can conclude that the different scenarios created mean values for the reproductive performance indicators that were dissimilar between the different scenarios. The mean values of the indicators for scenario 14, which is a scenario with average reproductive physiology and average management efficiency, compare well with the mean values of the traditionally used indicators for all 5020 herds in the Swedish DHI scheme in the year 2009 [17]. The herds in the DHI scheme had a CI of 408.7 days, CFI of 91 days and a CLI of 127 days. We have previously investigated how PV30 were distributed in 512 dairy herds in the DHI scheme. PV30 had a mean of 0.19 and an inter quartile range between 0.12 and 0.24 [25], which is in concordance with scenario 14. The results for worst case scenario, i.e. scenario 18, give reasonably similar numbers as for the worst herds in the Swedish DHI scheme [17].

**Table 3:** Mean and standard deviation for reproductive performance indicators for scenario 10-18 with a VWP of 79 days

Scenario		CI^a^	CFI^b^	CLI^c^	FI^d^	IC100^e^	NotIC- 200^f^	PV30^g^	IV30^h^
10	mean	387.9	75.6	97.7	57.5	0.35	0.28	0.40	0.70
	Std	4.2	3.6	5.4	9.5	0.05	0.14	0.05	0.04
11	mean	395.9	76.4	110.5	32.4	0.27	0.39	0.31	0.71
	Std	4.5	4	6.8	6.5	0.04	0.07	0.04	0.04
12	mean	404.3	75.8	119.6	20.5	0.21	0.44	0.25	0.71
	Std	6.4	4.1	8.9	6.8	0.05	0.05	0.05	0.05
13	mean	397.8	86	108	64.0	0.25	0.42	0.29	0.53
	Std	5	4.9	6.9	11.4	0.04	0.05	0.04	0.05
14	mean	410.5	88.5	121.4	39.7	0.17	0.47	0.21	0.52
	Std	6.6	5	7.4	8.3	0.04	0.05	0.04	0.04
15	mean	412	86.6	128.9	28.1	0.15	0.54	0.18	0.53
	Std	8	6.4	8.2	6.3	0.04	0.06	0.05	0.05
16	mean	414.9	94.1	110.9	86.5	0.15	0.50	0.18	0.34
	Std	7.9	7.5	8.7	15.8	0.04	0.06	0.04	0.05
17	mean	420.0	94.9	120.8	59.6	0.10	0.61	0.13	0.35
	Std	8.3	7	8.5	10.8	0.03	0.05	0.03	0.05
18	mean	422.0	94.9	123.6	52.6	0.09	0.67	0.11	0.35
	Std	12.2	6.9	9.6	12.6	0.03	0.05	0.03	0.05

^a^CI = Calving interval, days

^b^CFI = Calving to first service interval, days

^c^CLI = Calving to last service interval, days

^d^FI = fertility index

^e^IC100 = proportion of pregnant cows 100 days after calving

^f^NotIC200 = proportion of non pregnant cows 200 days after calving

^g^PV30 = proportion of pregnant cows 30 days after the herd VWP

^h^IV30 = proportion of inseminated cows 30 days after the herd VWP

When following management efficiency in descending order, from good to average to poor, i.e. starting with scenario 11 and comparing it to scenario 14 and 17, one can conclude that the descending management efficiency is reflected in the different reproductive performance indicators which all show impaired reproductive performance (Table 3). This is also true when following the reproductive physiology, from good to average to poor, i.e. starting with scenario 13 and comparing it to scenario 14 and 15. This shows that the performance indicators were affected by the simulated scenarios in the right and anticipated direction and gives credence to our evaluation of the reproductive performance indicators.

## Conclusion

Both the PV30 and the IV30 could be integrated in the reports that are provided to herds enrolled in the DHI. They would be useful in providing information to herd advisors, veterinarians and farmers on how the herd performs and for benchmarking against other herds. Research on actual herds must be done to establish the variation for these indicators and for investigating the optimal values. This must also be done to decide where to draw the line for when PV30 and IV30 should be considered too low and action is needed. Finally, when considering all aspects, i.e. assessing both reproductive management efficiency and reproductive physiology, ease of use, and preparedness for differences in management and future changes in management, the single best reproductive performance indicator seems to be PV30.

## Abbreviations

AUC: area under curve; CFI: calving to first insemination interval; CI: calving interval; CLI: calving to last insemination; CR:conception rate; DHI:dairy herd improvement scheme; ECM: energy corrected milk; FI: fertility index; IC100: InCalf 100 days after calving; IV30: percentage of inseminated cows after the voluntary waiting period plus 30 days; NotIC200: Not InCalf 200 days after calving; PC: pregnancy check; PV30: percentage of pregnant cows after the voluntary waiting period plus 30 days; ROC: receiver operating curve; SOMRS: Swedish official milk recording scheme; and VWP: voluntary waiting period.

## Competing interests

The authors declare that they have no competing interests.

## Authors' contributions

EL, HG and UE have performed this work as a part of EL's PhD-thesis. EL and UE were responsible for the simulation and statistical analyses. All authors have made contributions to interpretations and drawing of conclusions. EL managed the manuscript writing but all authors were involved in the preparation of the manuscript. All authors read and approved the final manuscript.
